# Zanubrutinib Combined With Rituximab in the Treatment of Bing‐Neel Syndrome: A Case Report

**DOI:** 10.1002/ccr3.71504

**Published:** 2025-12-01

**Authors:** Rongyao Zhang, Juan Chang, Yanqun Shen, Ping Hu, Lulu Zhang

**Affiliations:** ^1^ Department of Hematology, Taihe Hospital Hubei University of Medicine Shiyan Hubei People's Republic of China

**Keywords:** anti‐CD20 monoclonal antibody, Bing–Neel syndrome, flow cytometry, Waldenström macroglobulinemia, zanubrutinib

## Abstract

Bing‐Neel syndrome (BNS) is a rare complication of Waldenström macroglobulinemia, a condition with low incidence in clinical practice and prone to misdiagnosis. Vigilance is crucial for identifying this condition in patients with Waldenström macroglobulinemia presenting with neurological symptoms. In cases where a pathological biopsy is inconclusive, cerebrospinal fluid flow cytometry can be utilized to aid in diagnosis. Currently, there is no established standard treatment protocol for BNS, and treatment outcomes vary. In this study, a combination of zanubrutinib and R‐CHOP regimen was administered, with zanubrutinib being continued for the management of BNS, resulting in a notably favorable response.

**Summary**

- A 52‐year‐old male with lymphoplasmacytic lymphoma/Waldenström macroglobulinemia (LPL/WM) developed Bing‐Neel syndrome (BNS) following R‐CHOP chemotherapy.
- He received zanubrutinib 160 mg twice daily for 28 months, achieving complete symptom resolution, normalized laboratory parameters, and a very good complete response (VGCR).

## 1 Introduction

Waldenström's macroglobulinemia is a rare low‐grade non‐Hodgkin lymphoma originating from B cells, which predominantly affects elderly males with a median age of 64 years at diagnosis [1]. It represents about 2% of hematologic malignancies and presents with clinical features such as hyperviscosity syndrome, cytopenia, lymphadenopathy, and splenomegaly [1]. Cutaneous involvement in Waldenström's macroglobulinemia is uncommon. Bing–Neel syndrome (BNS) is an infrequent complication of Waldenström's macroglobulinemia characterized by clonal infiltration of lymphoplasmacytic cells into the central nervous system, occurring in approximately 1% of WM patients [2, 3]. The manifestations of Bing Neel syndrome vary widely and can include symptoms like headaches, cognitive impairment, weakness, and psychiatric manifestations. Here, we describe a case of a patient who developed Bing Neel syndrome as a secondary complication of Waldenström's macroglobulinemia and exhibited a remarkable response to Zanubrutinib, a second‐generation Bruton tyrosine kinase (BTK) inhibitor.

## 2 Case History

**FIGURE 1:**
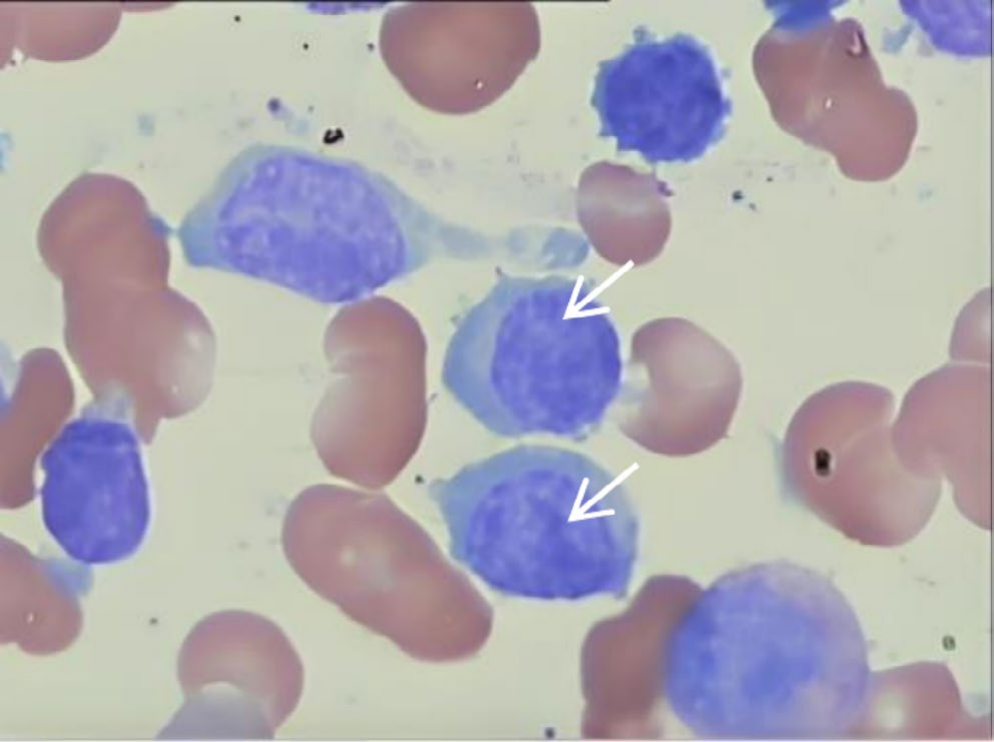
Bone marrow aspiration smear, The white arrows refer to Lymphomatoid Cells.

A 52‐year‐old male presented with a 2‐year history of pruritic erythematous papules and plaques on his trunk and elbows. Initially diagnosed as eczematous dermatitis, the condition persisted despite treatment with oral antihistamines, topical steroids, and immunomodulators (dapsone and cyclosporine). Recently, the pruritus intensified, prompting a referral to the hematology department due to a gradual decline in hemoglobin levels. Laboratory findings revealed mild anemia with a normal peripheral blood smear. Biochemical analysis showed total protein levels of 109.92 g/L, with albumin at 29.02 g/L and globulin at 80.9 g/L. Further investigations, including serum protein electrophoresis and immunofixation electrophoresis (IFE), identified an IgM kappa monoclonal protein (M‐protein) level of 3.2 g/dL, kappa light chain at 14510.5 mg/L, lambda light chain at 617 mg/L, and a kappa lambda light chain ratio of 23.52. A bone marrow examination revealed diffuse infiltration of Lymphoplasmacytoid Cells (LPCs) (Figure 1). The biopsy indicated the replacement of normal hematopoiesis by lymphoplasmacytic lymphoma, constituting 55% of the bone marrow cellularity.

**FIGURE 2:**
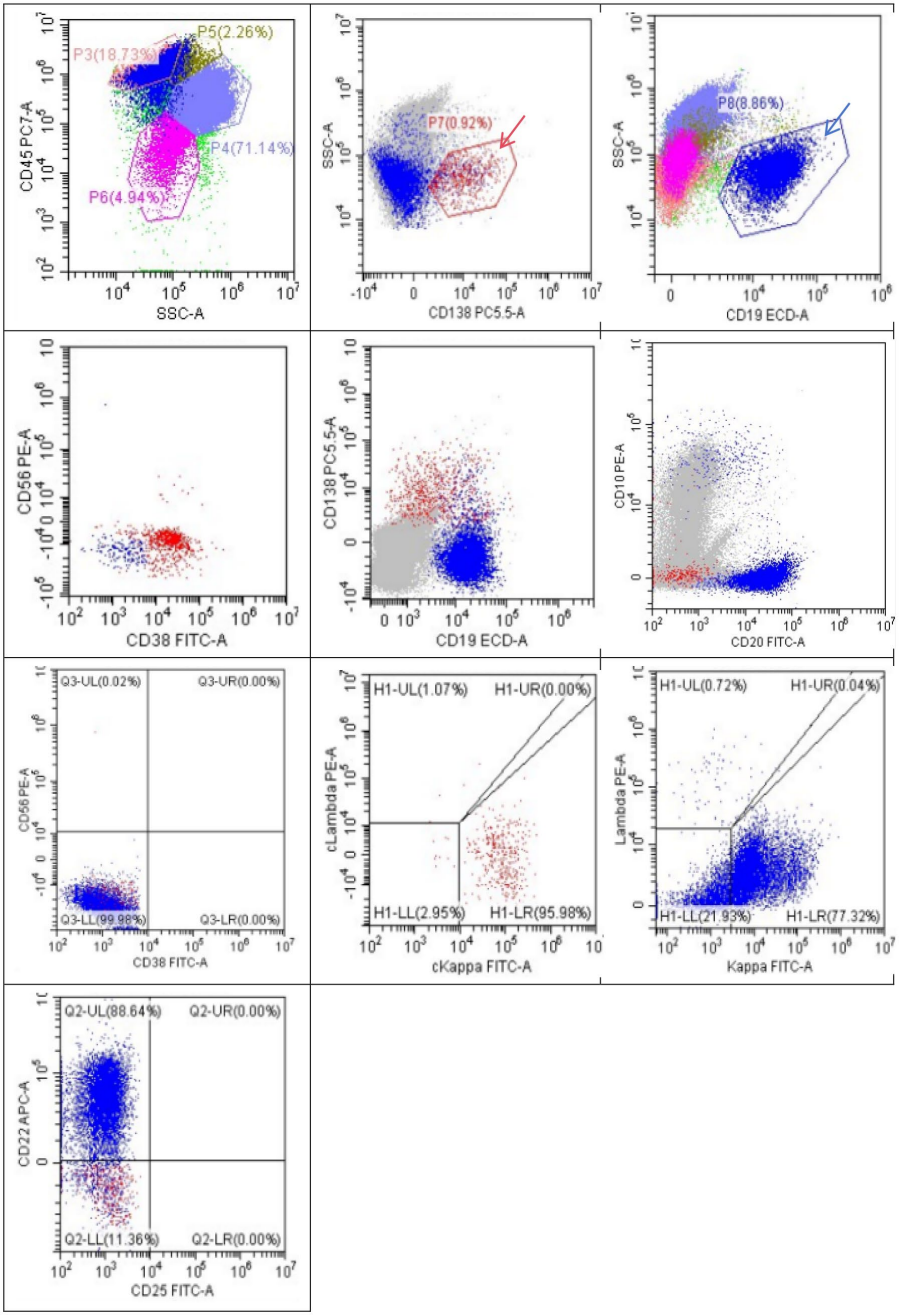
Flow cytometric analysis (FCM) showed that the abnormal plasma cells(The red arrow shows the area, about 0.92% of all nucleated cells in bone marrow) expressed CD38, CD138, cKaPPa, CD19; but not CD10 CD20 CD56 cLamba; abnormal B cells((The blue arrow shows the area, about 8.86% of all nucleated cells in bone marrow)) expressed CD19, CD20, CD22, HLA‐DR CD200 kappa but notCD10, CD38, CD23, CD5, CD25, FMC7, sIgM. The red dot plot represents abnormal plasma cell population, and the blue dot plot represents abnormal B cell population.

**FIGURE 3:**
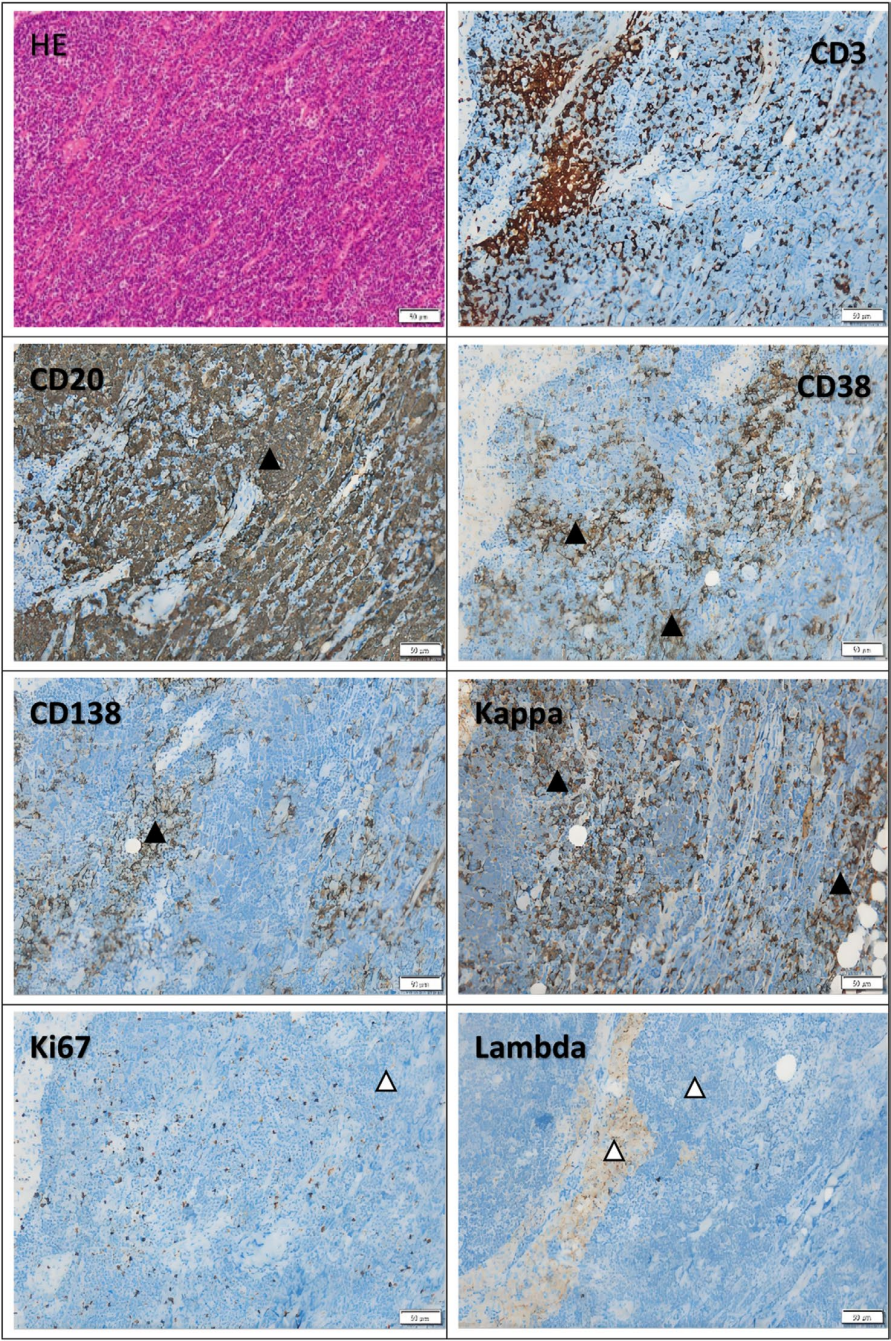
Biopsy of the right cervical lymph node showed non‐Hodgkin B‐cell lymphoma, low grade, and Immunohistochemistey indicated: CD20 (+), CD21 FDC net + (expand), CD3(−), CD5 (−), CD30 (−), Ki—67 (+ 10%), CyclinD1 (−), CD43 weak (+), Bcl‐2 + (weak), Bcl −6 (−), CD10 (−), MUM1 part (+), c—myc (In +), LEF1 (−), CD23 + (part), SOX—11 (−), CD79a (+), CD19 (+), CD38(+) CD56(−), kappa (+), lambda (−), CD138 part (+). In the figure, the light yellow region indicates weak positivity, exemplified by the region containing the white triangle. The brownish yellow region signifies moderate positivity, as observed in the vicinity of the black triangle. The blue areas are negative.

Analysis of bone marrow flow revealed that the CD138+ cell population constitutes approximately 0.92% of all nucleated cells. These cells express CD38 and cKappa, partially express CD19, and do not express CD10, CD20, CD56, or cLambda, indicative of monoclonal abnormal plasma cells. The CD19+ cell subset represents 8.86% of all nucleated cells and expresses CD20, CD22, and HLA‐DR, partially expresses CD200 and Kappa, and lacks CD10, CD38, CD138, CD5, CD23, FMC‐7, sIgM, and CD25, characterizing monoclonal abnormal mature B lymphocytes(Figure 2). 18F‐FDG PET‐CT imaging revealed mild FDG uptake in systemic bones, bilateral neck, axilla, mediastinum, groin, elbow fossa lymph nodes, and soft tissue shadows in shoulder dorsal, retroperitoneum, pelvic wall. A biopsy of the right cervical lymph node confirmed non‐Hodgkin B‐cell lymphoma, low grade, with immunohistochemical analysis showing positive staining for CD20, CD21, Bcl‐2, CD79a, CD19, CD38, kappa, lambda, and partial staining for CD23(Figure 3). In situ hybridization was negative for EBER. Molecular testing of the lymph node detected the MYD88 L265P gene mutation, while peripheral blood showed no MYD88 gene mutation but revealed a synonymous variation in CD79B Exon3 at c.366 T>C (p.C122=) in a heterozygous state. The patient was diagnosed with LPL/WM and underwent plasmapheresis along with 2 cycles of R‐CHOP chemotherapy comprising rituximab, cyclophosphamide, daunorubicin hydrochloride, vincristine, and prednisone. Following a 2‐week treatment period, the pruritic erythematous papules and plaques on the trunk and elbows showed resolution.

## 3 Differential Diagnosis, Investigations, and Treatment

**FIGURE 4:**
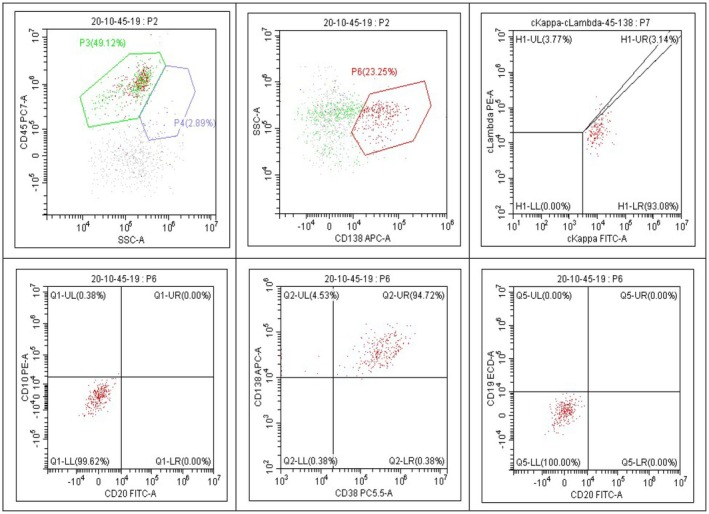
Flow cytometry of the CSF indicated that 23.25% of nucleated cells were CD138+ cells expressing CD38 and kappa, while not expressing CD20, CD10, CD19, or cLambda, indicative of a monoclonal aberrant plasma cell. The red dots in the picture represent abnormal cell populations.

During the third treatment session, the patient presented with a headache, facial numbness, and weakness, prompting suspicion of central nervous system infiltration. Subsequent cerebrospinal fluid (CSF) analysis revealed elevated protein levels (90 mg/dL) and an intracranial pressure of 320 mmH_2_O. Flow cytometry of the CSF indicated that 23.25% of nucleated cells were CD138+ cells expressing CD38 and kappa, while not expressing CD20, CD10, CD19, or cLambda, indicative of a monoclonal aberrant plasma cell. Additionally, 0.7% of nucleated cells were CD19+ cells expressing CD20 but not CD10, CD38, or CD138, with a Kappa/Lambda ratio of 0.92, showing no apparent phenotypic abnormalities (Figure 4). Droplet digital PCR detected an MYD88L265P mutation in the CSF. Peripheral blood flow cytometry showed no abnormalities. Despite declining a brain MRI, the patient was clinically diagnosed with BNS and continued on RCHOP and high‐dose methotrexate every 4 weeks. While symptoms improved gradually, intracranial pressure remained high, and bone marrow biopsy confirmed ongoing WM involvement (lymphoplasmacytic lymphoma cells comprising 40% of bone marrow cellularity). In January 2022, the patient initiated zanubrutinib at 160 mg twice daily, resulting in normalized intracranial pressure, and no malignant lymphocytes were detected in cytology or flow cytometry analysis. Within a week of zanubrutinib treatment initiation, the patient experienced clinical improvement with resolution of headache, facial numbness, and weakness.

## 4 Results (Outcome and Follow‐Up)

**FIGURE 5:**
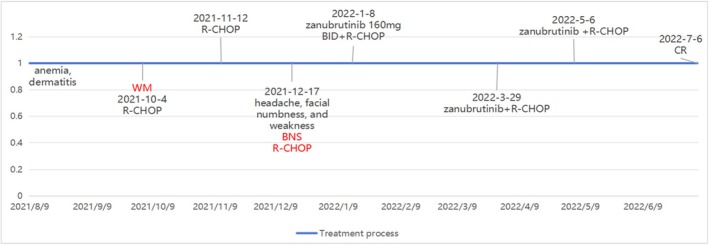
Changes in the patient's condition and important time node diagram. Timeline depicting the major events during the patient's disease course. BID, twice a day; BNS, Bing–Neel syndrome; CR, complete remission; R‐CHOP, rituximab, cyclophosphamide, hydroxydaunorubicin hydrochloride, vincristine, and prednisone; WM, Waldenström's macroglobulinemia. Dates were shown by year‐month‐day.

Upon 6 months of zanubrutinib therapy, the patient exhibited notable improvements in their hemoglobin levels, with a recovery to 124 g/dL, as well as in globulin, serum M‐protein, and IgM levels, which improved to 45.66 g/L, 560 mg/dL, and 6.3 g/L, respectively. A complete remission (CR) was confirmed by examination 7 months post initial BNS diagnosis (Figure 5). The patient continued a daily regimen of 160 mg zanubrutinib without encountering any adverse effects. At the time of reporting, the patient had been undergoing zanubrutinib treatment for 28 months and had sustained an IgM paraprotein level below 4 g/L, indicative of a very good complete response.

## 5 Discussion

Bing‐Neel syndrome is a rare complication of Waldenström's macroglobulinemia (WM), characterized by the infiltration of WM clonal plasma cells into the central nervous system [4]. First reported by Bing and von Neel in 1936, this syndrome has been documented in over 100 cases in the literature, representing less than 1% of all WM patients [5]. In a study across 8 French centers, 24 patients were diagnosed with BNS, with an average age of 62.4 years (range 35–80 years) and a male‐to‐female ratio of 9:1 [6]. BNS manifested as the initial symptom in 15%–36% of patients, occurring either concurrently with WM diagnosis or between 9 months and 24.7 years thereafter [7]. Notably, there have been no reported instances of BNS independent of WM.

The clinical presentation of BNS is varied and often nonspecific. Patients may report symptoms such as headache, nausea, vomiting, visual disturbances, hearing loss, paresthesias, pins and needles sensations, pain, seizures, or altered consciousness [5]. It is important to note that there is no specific clinical pattern or symptom that definitively confirms or rules out BNS. These symptoms typically progress gradually over weeks or months. The clinical manifestations of BNS are contingent upon the location and characteristics of the central nervous system infiltration. While abnormal brain and/or spinal MRI findings showing leptomeningeal or parenchymal disease can support the diagnosis of BNS [5], they alone are not sufficient for confirmation. A lack of abnormal MRI results does not negate the possibility of BNS. Therefore, the absence of MRI abnormalities should not be used as the sole basis for excluding the diagnosis of BNS [8].

Currently, the primary diagnostic method for BNS is the presence of WM cells in cerebrospinal fluid. If there is a central infiltration resembling a soft tissue mass, a biopsy of the affected region can confirm lymphoplasmacytic lymphoma [5]. Since obtaining brain or spinal cord tissue for clinical purposes is challenging, the detection of plasma cell infiltration in cerebrospinal fluid is essential for diagnosis. Cerebrospinal fluid flow cytometry is predominantly employed for clinical diagnosis and is capable of identifying atypical lymphocytes exhibiting plasma cell features.

Despite the patient's refusal to undergo a brain MRI following symptoms of headache and limb weakness, potentially impacting our diagnostic capabilities, we conducted a cerebrospinal fluid examination. This analysis revealed the presence of monoclonal abnormal plasma cells through cerebrospinal fluid flow cytometry, whereas peripheral blood flow cytometry yielded normal results. The detection of MYD88 (L265P) mutation in cerebrospinal fluid serves as valuable indicators for diagnosing BNS, although they are not considered diagnostic criteria [9]. Studies have indicated that over 90% of LPL/WM patients exhibit MYD88 gene mutations, which can serve as a relatively specific marker for LPL/WM [10]. Stephanie et al. demonstrated the presence of MYD88 mutations in the cerebrospinal fluid of BNS patients using real‐time quantitative PCR (QPCR) [9]. In this instance, the MYD88 gene mutation was detected in the cerebrospinal fluid. Subsequent to the diagnosis of LPL/WM, the patient developed headaches and increased intracranial pressure. Analysis of the cerebrospinal fluid revealed abnormal cloned plasma cells and numerous plasma cell‐like lymphocytes, in addition to a MYD88 gene mutation, thereby confirming the diagnosis of BNS.

A multi‐institutional international retrospective study reported a 59% estimated 3‐year survival rate for BNS patients [5]. The prognosis for BNS remains poor, with no standardized treatment currently available. The blood–brain barrier significantly influences drug efficacy in treating neurological and systemic conditions. Primary central nervous system lymphoma represents the main therapeutic approach for BNS, with intrathecal chemotherapy regimens typically including purine analogs (cladribine, fludarabine, bendamustine) and combinations of methotrexate with cytarabine [5]. Rituximab serves as a first‐line treatment for B‐cell malignancies, particularly Waldenström's macroglobulinemia (WM), where it has demonstrated substantial clinical efficacy. Multicenter retrospective studies have shown that rituximab improves overall survival in BNS patients [11, 12]. WM frequently harbors MYD88 L265P and CXCR4 mutations [10]. Bruton's tyrosine kinase (BTK) inhibitors have emerged as novel therapeutic agents for WM, demonstrating particular effectiveness in patients with MYD88 mutations.

Ibrutinib, a first‐generation Bruton's tyrosine kinase (BTK) inhibitor, is now considered a standard treatment for patients diagnosed with Waldenström macroglobulinemia (WM). In a phase 2 clinical trial involving 63 participants with relapsed or refractory WM, it was observed that 73% of the patients achieved a significant response, defined as at least a partial response (PR). The estimated two‐year progression‐free survival (PFS) rate was reported at 69% [13, 14]. Nonetheless, certain adverse events attributed to the non‐specific effects of ibrutinib, such as atrial fibrillation, bleeding episodes, and hypertension, were cited as causes for discontinuing the treatment [15].

Zanubrutinib, a potent BTK inhibitor, demonstrates lower off‐target inhibition compared to ibrutinib. Its pharmacokinetics, pharmacodynamics, and selectivity suggest potential for greater efficacy and improved safety over ibrutinib [14]. Studies have shown that zanubrutinib is highly effective in treating Waldenström macroglobulinemia (WM) and offers notable safety benefits, particularly in terms of cardiovascular toxicity. Zhang et al. [16] reported the distribution of zanubrutinib in the cerebrospinal fluid (CSF) for the first time, highlighting its effective and rapid penetration of the blood–brain barrier (BBB) with higher CSF concentrations than ibrutinib. The combination of zanubrutinib with high doses of methotrexate demonstrated significant clinical response and tolerability in primary central nervous system lymphoma patients [17]. Zanubrutinib has shown efficacy and safety in WM patients with central nervous system (CNS) infiltration, achieving an overall response rate of 73% [18, 19]. Becking et al. [18] suggested that zanubrutinib can achieve high response rates in patients with Bing‐Neel syndrome (BNS) with an acceptable toxicity profile. Therefore, zanubrutinib can be considered an effective and safe treatment option for BNS, both in initial and advanced disease stages.

We administered a chemotherapy regimen that involved combining an anti‐CD20 monoclonal antibody with a BTK inhibitor for this patient. Following four treatment cycles, the patient attained a partial response (PR) and subsequently continued oral Zanubrutinib maintenance therapy while receiving regular outpatient follow‐up.

## 6 Conclusion

In summary, the BNS is an uncommon complication of WM that poses diagnostic and therapeutic challenges. Close attention to the CSF analysis is crucial in WM patients; however, additional case validation is warranted.

## Author Contributions

**Rongyao Zhang:** conceptualization, data curation, formal analysis, investigation, methodology, writing – original draft, writing – review and editing. **Juan Chang:** conceptualization, data curation, formal analysis, methodology, writing – original draft, writing – review and editing. **Lulu Zhang:** supervision. **Ping Hu:** data curation. **Yanqun Shen:** supervision.

## Ethics Statement

All procedures performed in this study were in accordance with ethical standards of the Helsinki declaration.

## Consent

Written informed consent was obtained from the patient to publish this report in accordance with the journal's patient consent policy.

## Conflicts of Interest

The authors declare no conflicts of interest.

## Data Availability

All data generated during this study is included in this published article.
